# Statins use and prognosis in lung cancer patients treated with immune checkpoint inhibitors: evidence from a meta-analysis

**DOI:** 10.3389/fimmu.2026.1910678

**Published:** 2026-09-01

**Authors:** Dandan Liang, Qinghua Liu, Min Liu, Yi Li

**Affiliations:** 1 Department of Infectious Diseases, Chongqing Public Health Medical Center, Chongqing, China; 2 Department of General Surgery, Chongqing Public Health Medical Center, Chongqing, China; 3 Department of Oncology and Hematology, Liang Jiang Hospital of Chongqing Medical University, Chongqing, China

**Keywords:** immune checkpoint inhibitors, lung cancer, meta-analysis, prognosis, statins

## Abstract

**Background:**

Lung cancer remains one of the most common and lethal malignancies worldwide. The advent of immune checkpoint inhibitors (ICIs) has substantially improved survival outcomes in patients with advanced disease; nevertheless, a considerable proportion of patients exhibit suboptimal responses to ICI monotherapy. In recent years, statins have garnered attention for their potential immunomodulatory and anti-inflammatory properties. Preclinical evidence suggests that statins may enhance antitumor immune responses by modulating the tumor microenvironment and facilitating antigen presentation. However, existing clinical data remain inconclusive regarding whether statin use influences prognosis in lung cancer patients receiving ICIs. Accordingly, this meta-analysis was undertaken to evaluate the impact of statin use on survival outcomes in this patient population, with the aim of informing clinical decision-making regarding combination strategies.

**Methods:**

We systematically searched PubMed, Embase, the Cochrane Library, and Web of Science for clinical studies published from database inception to June 2026 that examined the association between statin use and outcomes in lung cancer patients treated with ICIs. Two investigators independently screened the literature, extracted data, and assessed the risk of bias using the Newcastle-Ottawa Scale (NOS). The primary endpoints were overall survival (OS) and progression-free survival (PFS). A meta-analysis was performed using Stata 15.0 software.

**Results:**

Twelve studies comprising 5,156 participants were included. Pooled analysis demonstrated that statin use was associated with significantly improved OS (HR = 0.76, 95% CI (0.63, 0.91), *P* = 0.003) and PFS (HR = 0.82, 95% CI (0.69, 0.96), *P* = 0.017). Subgroup analyses revealed that the survival benefit remained significant for both OS and PFS in multivariate-adjusted estimates. In univariate analyses and in larger studies (n > 500), the benefits did not attain statistical significance. Geographically, statins significantly improved OS in non-Asian populations, whereas the PFS benefit in these populations did not reach significance.

**Conclusions:**

This meta-analysis indicates that statin use is associated with significantly prolonged OS and PFS in lung cancer patients receiving ICIs, suggesting a potential role for statins in augmenting ICI efficacy. However, given that the included studies were predominantly retrospective observational designs, these findings should be interpreted with caution. Prospective, large-scale randomized controlled trials are warranted to confirm the survival benefits of combined statin and ICI therapy and to define the optimal timing and target population for such combination.

**Systematic review registration:**

https://www.crd.york.ac.uk/PROSPERO/, identifier CRD420261420311

## Introduction

Lung cancer is among the most common and deadliest malignant tumors globally, with over 2.2 million new cases and 1.8 million deaths annually. It remains the leading cause of cancer-related mortality in both China and worldwide (1, 2). Non-small cell lung cancer (NSCLC) accounts for approximately 85% of all lung cancers, and the majority of patients are diagnosed at locally advanced or metastatic stages, where conventional surgery, radiotherapy, and chemotherapy offer limited efficacy (3, 4). Over the past decade, immunotherapy, particularly immune checkpoint inhibitors (ICIs), has fundamentally transformed the treatment landscape for advanced lung cancer (5, 6). ICIs reinvigorate antitumor immunity by blocking programmed death receptor-1 (PD-1) and its ligand (PD-L1), or cytotoxic T-lymphocyte-associated antigen-4 (CTLA-4), thereby significantly prolonging patient survival (7, 8). Currently, pembrolizumab, nivolumab, atezolizumab, and other ICIs are recommended by domestic and international guidelines as the first-line or second-line standard therapies for advanced NSCLC without actionable driver mutations (9, 10).

Despite these breakthroughs, several challenges persist in clinical practice. First, only a minority of lung cancer patients derive durable benefit from ICI monotherapy, with primary and acquired resistance being common (11, 12). Second, immune-related adverse events (e.g., pneumonitis, colitis, rash) limit the broad applicability of ICIs (13, 14). Third, tumor-microenvironment factors such as immunosuppressive cells, metabolic dysregulation, and inflammatory signaling pathways can attenuate ICI efficacy (15, 16). Consequently, identifying combination strategies that enhance the antitumor activity of ICIs and improve patient prognosis has become a priority in cancer immunotherapy research.

Statins are widely prescribed for lipid regulation and for primary and secondary prevention of cardiovascular diseases (17, 18). In recent years, accumulating basic and preclinical evidence have revealed that statins possess pleiotropic effects beyond cholesterol lowering, including anti-inflammatory, antioxidant, immunomodulatory, and direct antiproliferative activities (19, 20). They can modulate immune cell subsets within the tumor microenvironment, for instance, by increasing CD8^+^ cytotoxic T-cell infiltration and reducing regulatory T cells (Tregs) and myeloid-derived suppressor cells (MDSCs) (21, 22). Furthermore, statins may upregulate MHC class I molecules and PD-L1 expression on tumor cells, potentially enhancing ICI-mediated recognition and attack (23, 24). On this mechanistic basis, combined statin and ICI therapy is hypothesized to exert synergistic antitumor effects.

However, clinical evidence regarding the prognostic impact of statins in lung cancer patients receiving ICIs remains conflicting. Several retrospective cohort studies have reported no significant improvement in overall survival (OS) or progression-free survival (PFS) with statin use (25–27), whereas others (21, 28) have demonstrated a 30%-40% reduction in mortality risk that persisted after multivariable adjustment. Prior meta-analyses have explored this question but have not reached a consensus (29, 30). Therefore, we conducted an updated meta-analysis of cohort studies to comprehensively evaluate the association between statin use and OS/PFS in lung cancer patients treated with ICIs, with the goal of providing evidence-based support for clinical combination strategies and for the design of future prospective randomized trials.

## Methods

This study was conducted in accordance with the updated Preferred Reporting Items for Systematic Reviews and Meta-Analyses (PRISMA) guidelines.

### Search strategy

We searched four major databases—PubMed, Embase, the Cochrane Library, and Web ofScience—from inception to June 2026. The search strategy combined MeSH terms and free-text keywords, including: “statin”, “HMG-CoA reductase inhibitor”, “atorvastatin”, “simvastatin”, “immune checkpoint inhibitors”, “PD-1 inhibitor”, “PD-L1 inhibitor”, “pembrolizumab”, “nivolumab”, “atezolizumab”, “lung cancer”, “non-small cell lung cancer”, “NSCLC”, “small cell lung cancer”, “SCLC”, “overall survival”, “progression-free survival”, etc. In addition, we manually screened the reference list of included studies to identify further potentially relevant publications. The detailed search strategies for each database are provided in **Supplementary Table 1**.

### Inclusion and exclusion criteria

Studies were eligible if they met all of the following criteria:

(1) Patients with pathologically confirmed lung cancer receiving ICIs treatment; (2) exposure to statins during or prior to the treatment period; (3) Reported outcomes of OS or PFS with hazard ratios (HRs) and 95% confidence intervals (CIs); and (4) cohort study design.

Studies were excluded if they met any of the following:

(1) Conference abstracts, reviews, case reports, animal experiments or cell-based studies; (2) Duplicate publications with incomplete data; (3) Studies for which full texts could not be obtained and authors could not be contacted; or (4) Survival data that did not clearly distinguish between statin users and non-users.

### Data extraction

Two investigators independently screened titles and abstracts to exclude irrelevant records, then reviewed full texts of the remaining articles for final inclusion. Disagreements were resolved through discussion. A standardized data extraction form was used to collect: first author, publication year, country, study design, sample size, mean age, proportion of males, type of ICIs, timing of statin use, median follow-up, and outcome data.

### Risk of bias assessment

The Newcastle-Ottawa Scale (NOS) was used to assess the risk of bias in included cohort studies. This scale comprises three domains: population selection (4 items, 4 stars), inter-group comparability (1 item, 2 stars), and outcome assessment (3 items, 3 stars), with a maximum of 9 stars. Studies were categorized as low (0–4 stars), moderate (5–6 stars), or high (7–9 stars) quality. Two reviewers independently scored each study and resolved discrepancies by consensus.

### Statistical analysis

Meta-analyses were performed using Stata 15.0. Pooled HRs with 95% CIs were calculated for OS and PFS. When HRs were not directly reported, they were estimated from Kaplan–Meier curves and follow-up data using the method described by Tierney et al. Heterogeneity was assessed using the Q test (*P* < 0.1 indicating significant heterogeneity) and the I^2^ statistic (I^2^ < 25%: low; 25%–75%: moderate; >75%: high). A fixed-effects model was applied when I^2^ < 50% and *P* > 0.1 (31); otherwise, a random-effects model was employed (32). Predefined subgroup analyses were performed to explore potential sources of heterogeneity: (1) by HR source (univariate vs. multivariate); (2) by sample size (<500 vs. >500); and (3) by region (Asian vs. non-Asian). In each subgroup, HRs were pooled using a random-effects model, and between-group differences were compared. Sensitivity analyses were conducted by sequentially excluding individual studies to assess the robustness of the pooled estimates. Publication bias was evaluated using Egger’s test, Begg’s test, and funnel plots.

## Results

### Search results

**Figure 1:**
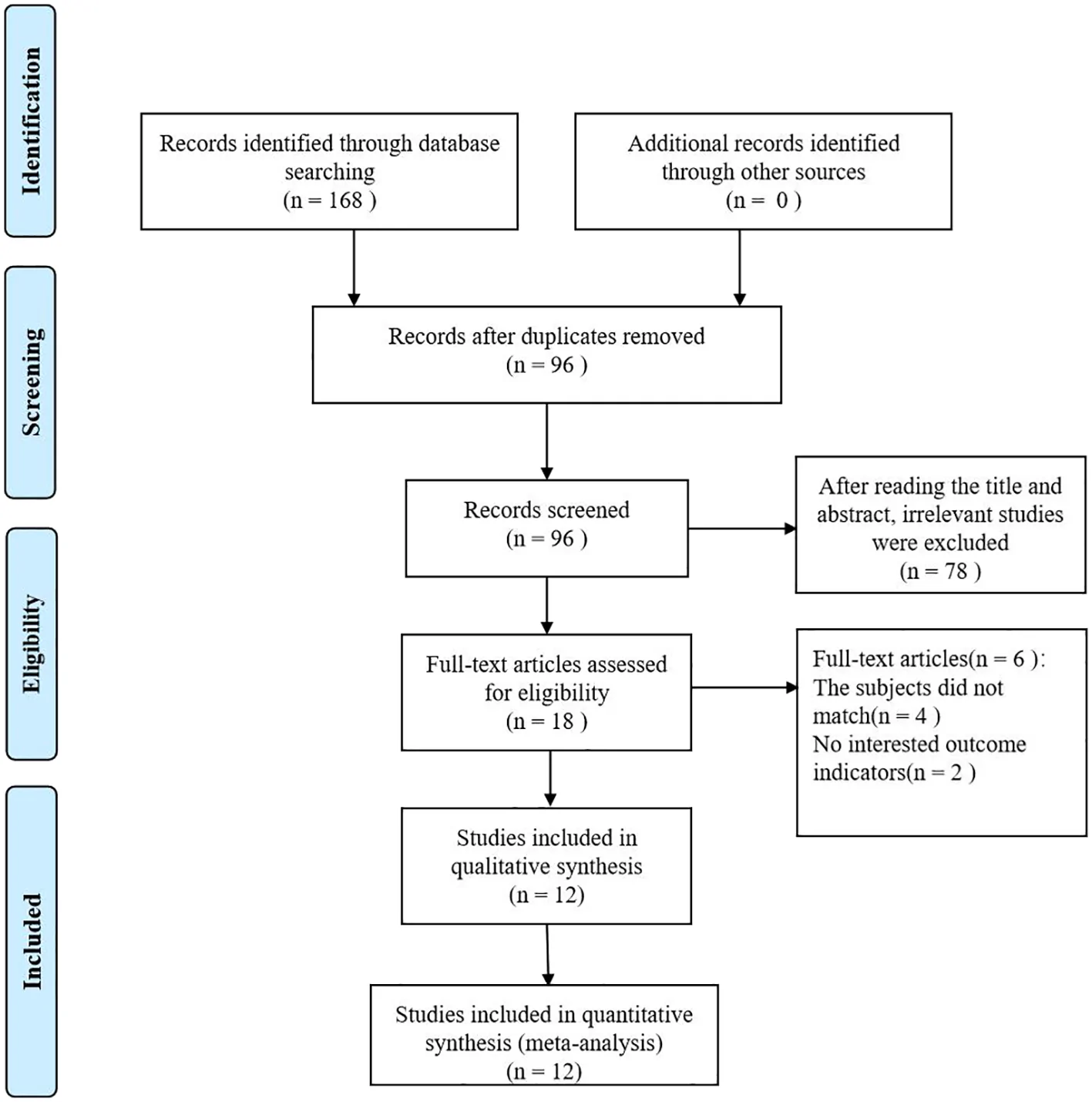
Flowchart of study selection.

The initial search yielded 168 records. After removing duplicates using EndNote, 96 remained. Screening titles and abstracts excluded 78 records (irrelevant topics, reviews, conference abstracts, case reports, animal experiments, or non-original studies). Full-text assessment of the remaining 18 articles led to the exclusion of 6 that did not meet eligibility criteria. Ultimately, 12 studies (21, 25–28, 33–39) were included in the meta-analysis (**Figure 1**).

### Characteristics of included studies

The baseline characteristics of the included studies are summarized in **Table 1**. The 12 studies were published between 2019 and 2026 and originated from Europe (Spain, Italy, France, the Netherlands, the Czech Republic), Asia (Japan, China), and North America (the United States). Italy contributed the largest number of studies, Japan had two, and each of the other countries contributed one. In terms of design, one study was a prospective cohort, and the remaining 11 were retrospective cohorts. Sample sizes ranged from 67 to 1,401. Mean patient age ranged from 63.7 to 74.1 years, and the proportion of males ranged from 52% to 87%. All studies used anti-PD-1 or anti-PD-L1 antibodies as ICI therapy. Statin use was defined as prior to or during ICI treatment. Follow-up durations varied widely (9.0–21.8 months), with four studies not reporting specific follow-up times. Eleven studies reported OS, nine reported PFS, and eight reported both. NOS scores ranged from 7 to 9, indicating high methodological quality (**Table 2**).

**Table 1:** The baseline characteristics of included studies.

Study	Country	Study design	Sample size	Mean age (years)	Male (%)	ICIs used	Statin used	Follow-up period(Months)	Outcomes
Callejo et al., 2021 (33)	Spain	Retrospective cohort study	262	63.7	69%	Anti-PD-1/PD-L1 antibodies	Before ICI treatment	Median OS:11.1	OS,PFS
Cantini et al., 2021 (28)	Italy/Netherlands	Retrospective cohort study	179	NR	NR	Anti-PD-1antibodies	Before ICI treatment	Median OS:17.0	OS,PFS
Cortellini et al., 2021 (34)	Italy/UK	Retrospective cohort study	950	70.1	66%	Anti-PD-1antibodies	Before ICI treatment	Median OS:21.8	OS,PFS
Kostine et al., 2021 (35)	France	Retrospective cohort study	635	64.5	70%	Anti-PD-1/PD-L1 antibodies	Before ICI treatment	Median OS:16.0	OS
Marrone et al., 2025 (36)	US	Retrospective cohort study	1401	74.1	52%	Anti-PD-L1 antibodies	Before or during ICI treatment	Median OS:9.0	OS
Miura et al., 2021 (37)	Japan	Retrospective cohort study	300	65	75%	Anti-PD-1antibodies	Before ICI treatment	Median OS:11.7	OS
Omori et al., 2019 (38)	Japan	Prospective cohort study	67	67	69%	Anti-PD-1antibodies	Before ICI treatment	NR	OS
Rossi et al., 2021 (25)	Italy	Retrospective cohort study	122	71	87%	Anti-PD-1/PD-L1 antibodies	Before ICI treatment	NR	OS,PFS
Svaton et al., 2020 (26)	Czech Republic	Retrospective cohort study	224	67	59%	Anti-PD-1antibodies	Before or during ICI treatment	Median PFS:5.9	OS,PFS
Takada et al., 2022 (27)	Japan	Retrospective cohort study	390	67	79%	Anti-PD-1antibodies	Before ICI treatment	Median OS:13.7	OS,PFS
Yakobson et al., 2026 (39)	Israel	Retrospective cohort study	391	67.4	69%	Anti-PD-1antibodies	Before ICI treatment	NR	OS,PFS
Yang et al., 2025 (21)	China	Retrospective cohort study	235	69	86%	Anti-PD-1/PD-L1 antibodies	Before ICI treatment	NR	OS,PFS

ICI, immune checkpoint inhibitor; OS, overall survival; PFS, progression-free survival; NR, not reported; OS, overall survival; PD-1, programmed death receptor 1; PD-L1, programmed death ligand 1.

**Table 2:** Risk of bias assessment.

Author (year)	Selection	Comparability	Outcome	Total score
Callejo et al., 2021 (33)	★★★	★★	★★★	8
Cantini et al., 2021 (28)	★★★	★★	★★★	8
Cortellini et al., 2021 (34)	★★★	★★	★★★	8
Kostine et al., 2021 (35)	★★★	★★	★★★	8
Marrone et al., 2025 (36)	★★★	★★	★★★	8
Miura et al., 2021 (37)	★★★★	★★	★★★	9
Omori et al., 2019 (38)	★★★	★	★★★	7
Rossi et al., 2021 (25)	★★★	★	★★★	7
Svaton et al., 2020 (26)	★★★	★	★★★	7
Takada et al., 2022 (27)	★★★	★★	★★★	8
Yakobson et al., 2026 (39)	★★★	★	★★★	7
Yang et al., 2025 (21)	★★★	★★	★★★	8

### Impact of statins on overall survival

**Figure 2:**
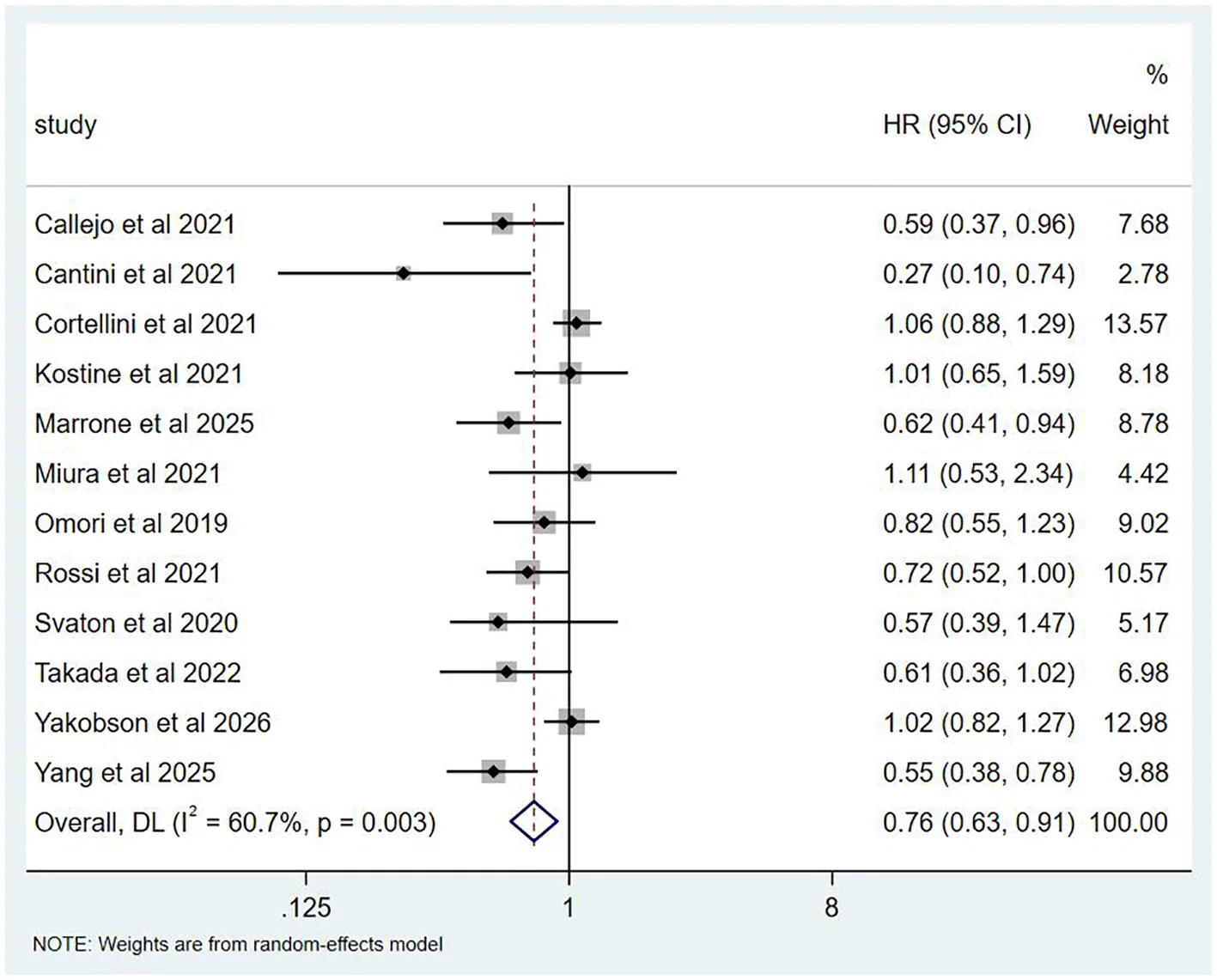
The impact of statin on the overall survival of lung cancer patients treated with immune checkpoint inhibitors.

Twelve studies evaluated the association between statin use and OS. Heterogeneity was moderate (I² = 60.7%, *P* = 0.003), prompting the use of a random-effects model. Pooled analysis showed that statins significantly improved OS (HR = 0.76, 95% CI: 0.63–0.91, *P* = 0.003) (**Figure 2**).

### Impact of statins on progression-free survival

**Figure 3:**
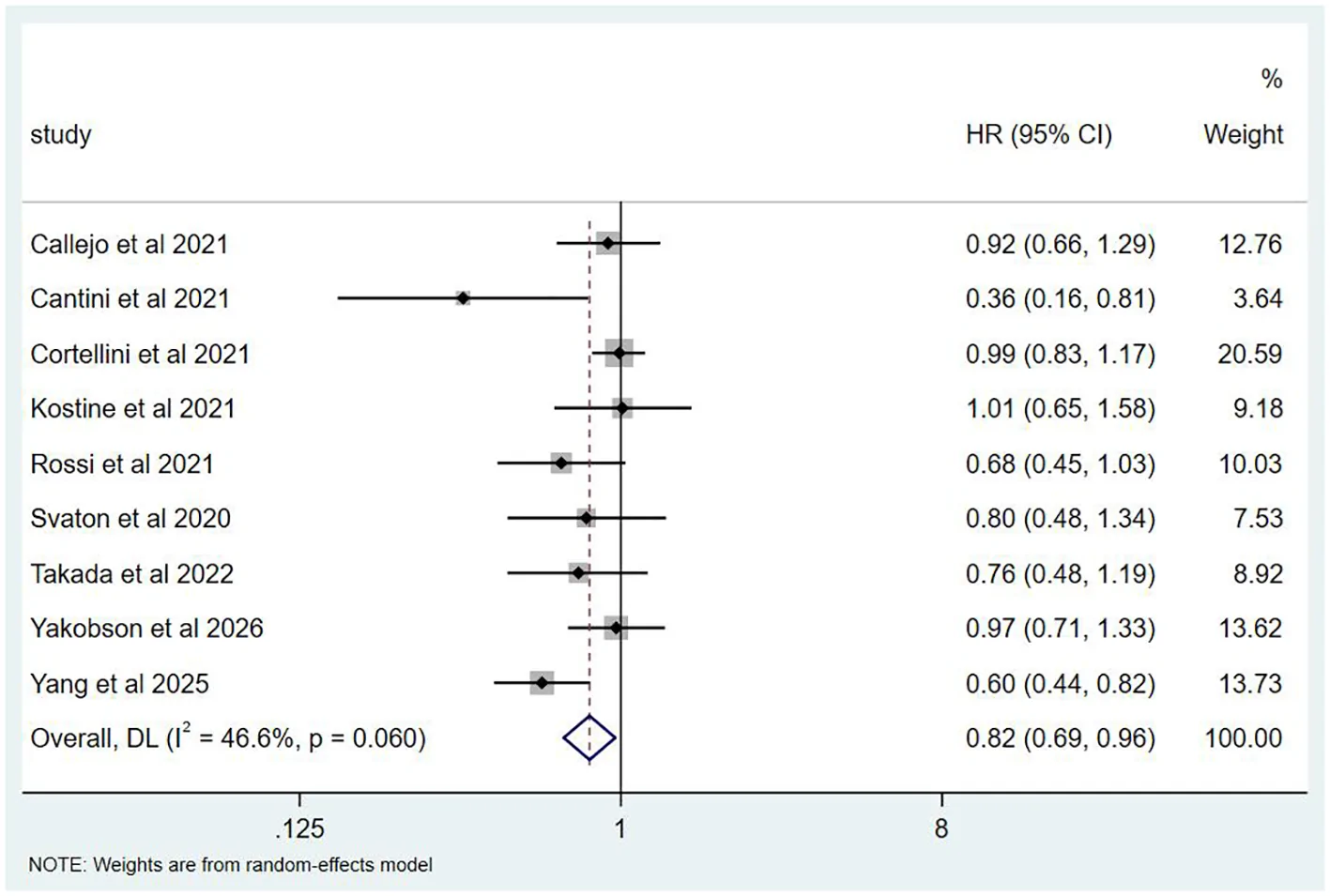
The impact of statin on the progression free survival of lung cancer patients treated with immune checkpoint inhibitors.

Nine studies assessed the association between statin use and PFS. Heterogeneity was moderate (I² = 46.6%, *P* = 0.060), and a random-effects model was applied. Statin use was associated with significantly improved PFS (HR = 0.82, 95% CI: 0.69–0.96, *P* = 0.017) (**Figure 3**).

### analyse Subgroup s

To explore the potential sources of heterogeneity, subgroup analyses were performed based on HR source, sample size, and region (**Table 3**).

**Table 3:** Subgroup analysis of each outcomes.

Subgroup analysis	No. of studies	Heterogeneity		HR (95%CI)	P value
		I^2^	P		
>OS					
Source of HR					
Univariate	7	41.60%	0.113	0.84 (0.69, 1.02)	s0.085
Multivariate	5	74.80%	0.003	**0.63 (0.43, 0.92)**	**0.018**
Sample size					
< 500	9	55.20%	0.022	**0.70 (0.56, 0.88)**	**0.002**
> 500	3	62.50%	0.07	0.90 (0.65, 1.24)	0.516
Region					
Asian	4	25.20%	0.26	**0.69 (0.53, 0.91)**	**0.008**
Non-Asian	8	64.30%	0.007	**0.78 (0.63, 0.97)**	**0.029**
>PFS					
Source of HR					
Univariate	6	0%	0.551	0.92 (0.82, 1.05)	0.217
Multivariate	3	67.10%	0.048	**0.65 (0.42, 0.99)**	**0.048**
Sample size					
< 500	7	35.90%	0.154	**0.75 (0.62,0.91)**	**0.004**
> 500	2	0.00%	0.934	0.99 (0.85, 1.16)	0.927
Region					
Asian	2	0.00%	0.4	**0.65 (0.50, 0.84)**	**0.001**
Non-Asian	7	29.00%	0.207	0.89 (0.75, 1.04)	0.138

Bold text indicates that the differences are statistically significant.

OS subgroup analyses: When HRs were derived from univariate analyses, the OS difference between statin users and non-users was not statistically significant (HR = 0.84, 95% CI: 0.69–1.02, *P* = 0.085). In contrast, multivariate-adjusted HRs showed a significant OS benefit (HR = 0.63, 95% CI: 0.43–0.92, *P* = 0.018). In studies with sample sizes <500, statins significantly improved OS (HR = 0.70, 95% CI: 0.56–0.88, *P* = 0.002), whereas in studies with >500 patients, the benefit was not significant (HR = 0.90, 95% CI: 0.65–1.24, *P* = 0.516). By region, statins significantly improved OS in both Asian (HR = 0.69, 95% CI: 0.53–0.91, *P* = 0.008) and non-Asian populations (HR = 0.78, 95% CI: 0.63–0.97, *P* = 0.029).

PFS subgroup analyses: For PFS, univariate-derived HRs did not show a significant difference (HR = 0.92, 95% CI: 0.82–1.05, *P* = 0.217), whereas multivariate-adjusted HRs indicated a significant benefit (HR = 0.65, 95% CI: 0.42–0.99, *P* = 0.048). In studies with <500 patients, statins significantly improved PFS (HR = 0.75, 95% CI: 0.62–0.91, *P* = 0.004), but in larger studies (>500), the benefit was not significant (HR = 0.99, 95% CI: 0.85–1.16, *P* = 0.927). Regionally, statins significantly improved PFS in Asian populations (HR = 0.65, 95% CI: 0.50–0.84, *P* = 0.001), whereas the PFS benefit in non-Asian populations did not reach significance (HR = 0.89, 95% CI: 0.75–1.04, *P* = 0.138).

### Sensitivity analysis

**Figure 4:**
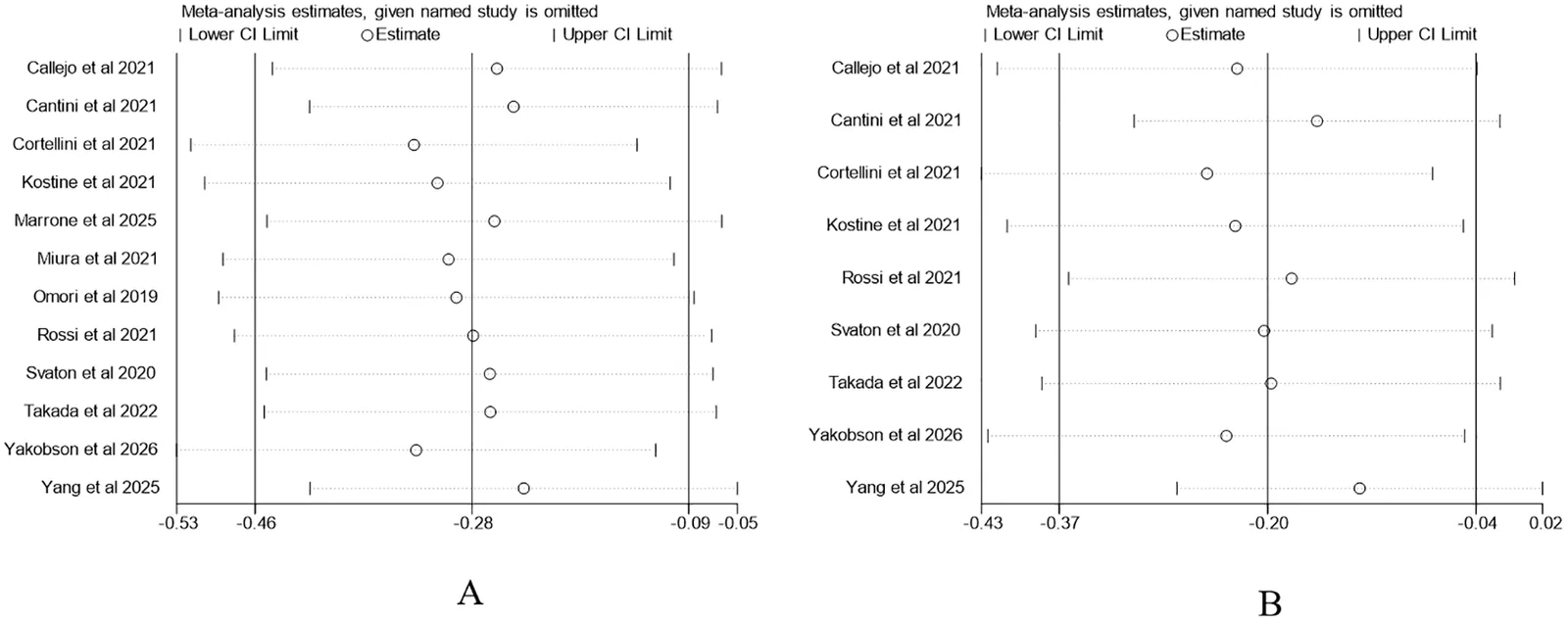
Sensitivity analysis: **(A)** overall survival; **(B)** progression-free survival.

Leave-one-out sensitivity analyses for both OS and PFS confirmed that no single study disproportionately influenced the pooled estimates (**Figure 4**).

### Publication bias

**Figure 5:**
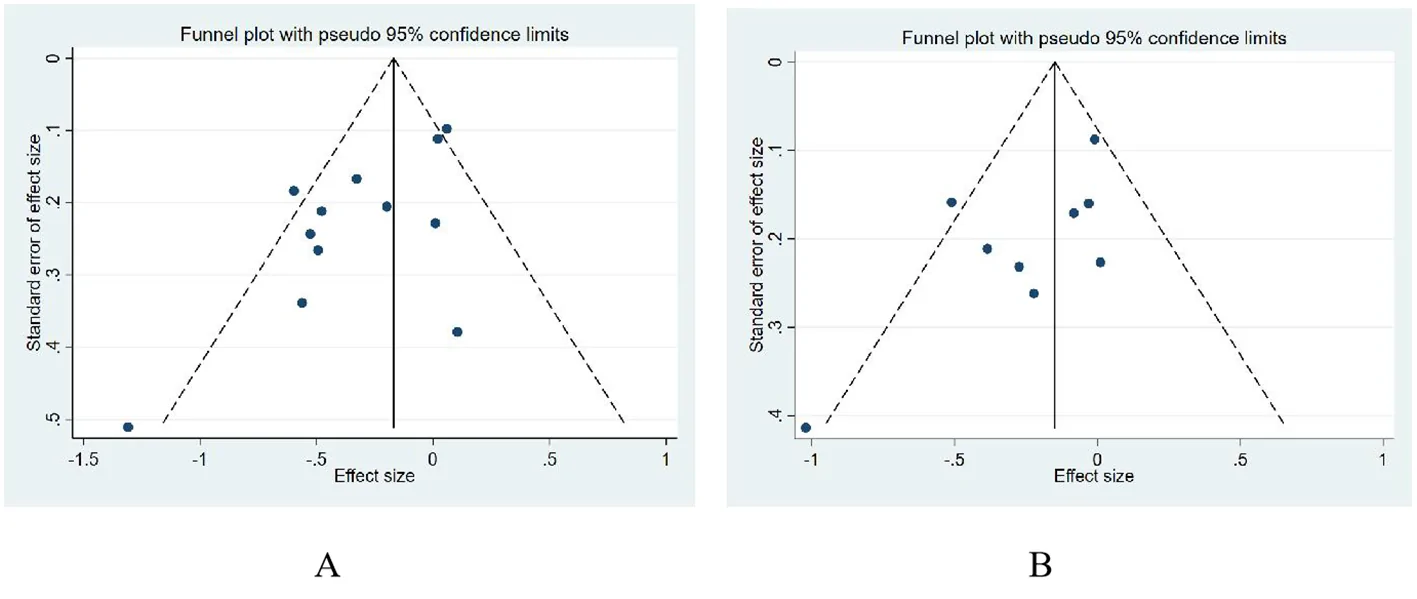
Funnel plot: **(A)** overall survival; **(B)** progression-free survival.

The funnel plot exhibited some asymmetry, suggesting possible publication bias (**Figure 5**). However, Egger’s test and Begg’s test did not indicate statistically significant publication bias (**Table 4**).

**Table 4:** Publication bias analysis.

Outcome	Egger’s tests (*P* Value)	Begg’s tests (*P* Value)
Overall survival	0.223	0.067
Progression-free survival	0.355	0.086

## Discussion

This meta-analysis incorporated 12 cohort studies comprising 5,156 lung cancer patients receiving ICI therapy. To our knowledge, it represents the largest and most current synthesis on this topic, including the most recently published data and providing a comprehensive evaluation of the association between statin use and survival outcomes. Our findings indicate that statins significantly improve OS and PFS. However, the statistical significance of these benefits varied across subgroups. In univariate analyses and in studies with >500 patients, neither OS nor PFS reached statistical significance. Geographically, statins significantly improved OS in non-Asian populations, but the PFS benefit in these populations did not attain significance. This discrepancy between OS and PFS in non-Asian cohorts may reflect differences in statistical power, event counts, or the influence of post-progression therapies on OS.

Notably, prior meta-analyses have yielded inconsistent results. A 2022 meta-analysis including seven studies found no OS or PFS benefit for statins in NSCLC patients treated with ICIs (30), a conclusion potentially constrained by the included studies’ publication dates (up to 2021), relatively small sample sizes, and underrepresentation of Asian populations. Another pan-cancer meta-analysis by Liao et al. (2025), which included 25 studies and 46,154 patients across multiple tumor types, demonstrated overall OS and PFS benefits for statins, but the NSCLC-specific subgroup analysis did not show a significant survival advantage (29). However, the recent publication of two large, high-quality retrospective studies by Yang et al. and Marrone et al. has meaningfully updated the evidence base (21, 36). Yang et al. observed significant survival benefits even after propensity score matching (21), and Marrone et al. reported similar findings after multivariable adjustment (36). Although both studies remain retrospective by design, their methodological rigor provides strong external support for our positive findings. Collectively, as the number of included studies, sample size, and confounding control have increased, the survival benefit of statins in lung cancer patients receiving ICIs has become evident in the most recent meta-analyses, suggesting that earlier negative meta-analyses may have been limited by insufficient statistical power and outdated evidence. Our study incorporated the latest cohort data, overturning previous negative conclusions. For instance, Song et al. (40) demonstrated that in lung cancer patients with cardiovascular risk factors but without prior cardiovascular events, statin use during immunotherapy was associated with reduced major adverse cardiovascular events and all-cause mortality, without increasing serious adverse events. Beyond updating the evidence, our study expanded the sample to 12 studies and, for the first time, systematically compared univariate versus multivariate-adjusted estimates. Moreover, previous meta-analyses rarely conducted subgroup analyses by region or sample size; our study fills this gap and reveals a greater benefit in Asian populations.

In multivariate analyses, the OS benefit of statins was more pronounced, whereas univariate analyses did not reach significance. This discrepancy underscores the importance of adjusting for potential confounders (e.g., age, sex, performance status, smoking history, concomitant medications), which may otherwise obscure a true protective effect. Multivariate adjustment revealed the genuine survival benefit of statins, reinforcing the need for rigorous confounding control in future prospective studies. The observation that small-sample studies showed significant statin benefits while larger studies did not warrants careful interpretation. On one hand, effect sizes in small studies may be overestimated; on the other hand, larger studies are often multicenter and higher in quality, and their null results may more closely reflect real-world practice. Nevertheless, the OS HR point estimate in the large-sample subgroup was 0.77 (with 95% CI crossing 1), and the direction remained favorable for statins, suggesting that increasing sample size may widen confidence intervals and that statistical power could be limited by between-group baseline imbalances and heterogeneity in definitions of statin exposure.

The OS and PFS benefits of statins were significantly greater in Asian populations than in non-Asian populations. This difference may be attributable to genetic background, dietary patterns, polymorphisms in statin-metabolizing enzymes (e.g., CYP450), tumor mutational burden, and racial variations in ICI efficacy. It is well established that the driver mutation spectrum (e.g., EGFR, ALK) differs substantially between Asian and non-Asian NSCLC patients, and the overall response rate to ICIs may vary accordingly. Additionally, the types and doses of statins used in Asian studies (e.g., rosuvastatin, pitavastatin) may differ from those used in non-Asian settings. Cross-ethnic comparative studies are needed to confirm this finding.

The potential mechanisms by which statins enhance ICI efficacy are multifaceted. First, statins inhibit HMG-CoA reductase, blocking the mevalonate pathway and thereby preventing the isoprenylation of small GTPases such as Ras, Rho, and Rac. This suppresses tumor cell proliferation, induces apoptosis, and promotes autophagy (41, 42). Recent studies have shown that low expression of mevalonate-pathway genes correlates with increased CD8^+^ T-cell infiltration and improved OS, and that lipophilic statins can induce ferroptosis via GPX4 depletion, synergizing with anti-PD-1 antibodies (43). Second, statins exert important immunomodulatory effects on the tumor microenvironment. Mao et al. demonstrated in NSCLC models that statins can convert an “immune-cold” tumor microenvironment into an “inflammatory” phenotype, thereby enhancing ICI responsiveness (22). Animal studies have shown that statins reduce MDSC and Treg infiltration while promoting CD8^+^ cytotoxic T-lymphocyte recruitment and activation, thus reversing immunosuppression (44, 45). Third, statins regulate the expression of immune-related molecules on tumor cells. Tilkin-Mariamé et al. found that statins enhance IFN-γ-induced MHC class I antigen expression (23)and induce CD80 and CD86 co-stimulatory molecule expression (23). Notably, recent molecular studies have shown that statins actually suppress PD-L1 expression (22, 46), suggesting that statins may sensitize tumors to ICIs by reducing PD-L1-mediated immune evasion rather than by upregulating PD-L1. Fourth, the anti-inflammatory effects of statins (e.g., inhibition of the NF-κB pathway and reduction of IL-6 and TNF-α levels) may mitigate ICI-related immune adverse events, indirectly improving treatment adherence and survival prognosis (19, 47).

This study has several limitations that warrant caution in interpreting the results. First, the majority of included studies were retrospective cohorts, inherently susceptible to selection and information biases, and residual confounding cannot be entirely excluded. Second, definitions of statin use varied across studies (some restricted to pre-treatment use, others including use during treatment), and types (lipophilic vs. hydrophilic) and intensities were not distinguished, potentially affecting effect estimates. Third, subgroup analyses revealed that univariate estimates for both OS and PFS did not reach statistical significance, whereas multivariate estimates did, indicating that unadjusted confounding may bias effect estimates toward the null. However, the sets of adjusted covariates were not consistent across studies, and residual confounding may persist. Fourth, funnel plot asymmetry suggests possible publication bias, with positive results more likely to be published, potentially overestimating the true pooled effect. Finally, the retrospective studies included in this study all defined statin exposure by comparing “users” with “non-users”. Such a design inherently carries the risk of immortal-time bias, where patients in the exposure group must survive until the start of exposure before they can start taking the medication, which may lead to an overestimation of the survival benefits. Although some original studies mitigated this bias to some extent by using time-dependent covariate models or propensity score matching, not all studies did so. Therefore, the aggregated results should be interpreted with caution. Future prospective randomized controlled trials can fundamentally eliminate this bias by synchronizing the exposure design.

Despite these limitations, our study provides important evidence-based guidance for clinical practice. In lung cancer patients receiving ICI therapy, statin use may confer survival benefits, particularly in Asian populations. Given their low cost, oral convenience, favorable safety profile, and widespread use for cardiovascular primary prevention, statins represent a pharmacoeconomically attractive combination partner for ICIs. However, before routine combination therapy can be recommended, prospective randomized controlled trials are needed to clarify: (1) the optimal timing of statin initiation (prior to or concurrent with ICIs); (2) the most appropriate type and dose of statin; and (3) which patient subgroups derive the greatest benefit (e.g., based on PD-L1 expression, tumor mutational burden, or statin-metabolizing genotype).

## Conclusion

This meta-analysis indicates that in lung cancer patients treated with ICIs, statin use is associated with significantly improved OS and PFS, suggesting that statins may enhance ICI efficacy. Given that the included studies were predominantly retrospective observational designs, these findings should be interpreted with caution. Prospective, large-sample randomized controlled trials are warranted to confirm the survival benefits of combined statin and ICI therapy and to determine the optimal timing and target population for such combination.

## Data Availability

The datasets presented in this study can be found in online repositories. The names of the repository/repositories and accession number(s) can be found in the article/**Supplementary Material**.

## Supplementary material

Supplementary Table 1 Search strategy.

## References

[1] Sung H Ferlay J Siegel RL Laversanne M Soerjomataram I Jemal A . Global cancer statistics 2020: Globocan estimates of incidence and mortality worldwide for 36 cancers in 185 countries. CA: A Cancer J For Clin. (2021) 71:209–49. doi: 10.3322/caac.21660 33538338

[2] Cao W Chen HD Yu YW Li N Chen WQ . Changing profiles of cancer burden worldwide and in China: A secondary analysis of the global cancer statistics 2020. Chin Med J. (2021) 134:783–91. doi: 10.1097/cm9.0000000000001474 33734139 PMC8104205

[3] Li N Zheng X Gan J Zhuo T Li X Yang C . Effects of glucocorticoid use on survival of advanced non-small-cell lung cancer patients treated with immune checkpoint inhibitors. Chin Med J. (2023) 136:2562–72. doi: 10.1097/cm9.0000000000002544 37925595 PMC10617908

[4] Padinharayil H Varghese J John MC Rajanikant GK Wilson CM Al-Yozbaki M . Non-small cell lung carcinoma (Nsclc): Implications on molecular pathology and advances in early diagnostics and therapeutics. Genes Dis. (2023) 10:960–89. doi: 10.1016/j.gendis.2022.07.023 37396553 PMC10308181

[5] Han J Yang Z Zhao H . Lung cancer immunotherapy in 2025: Where we stand and what comes next? Front Immunol. (2025) 16:1728163. doi: 10.3389/fimmu.2025.1728163 41624891 PMC12855455

[6] Zhou CY Qi YF Li HJ Zhang C Lin BX Lin JT . Current and future immunotherapies for Nsclc. J Hematol Oncol. (2026) 19:28. doi: 10.1186/s13045-026-01791-w 42057114 PMC13130492

[7] Raskova Kafkova L Mierzwicka JM Chakraborty P Jakubec P Fischer O Skarda J . Nsclc: From tumorigenesis, immune checkpoint misuse to current and future targeted therapy. Front Immunol. (2024) 15:1342086. doi: 10.3389/fimmu.2024.1342086 38384472 PMC10879685

[8] Wolf E Sacchi de Camargo Correia G Li S Zhao Y Manochakian R Lou Y . Emerging immunotherapies for advanced non-small-cell lung cancer. Vaccines. (2025) 13:128. doi: 10.3390/vaccines13020128 40006675 PMC11861325

[9] Pu X Zhou Y Wang J Wu L . Immune checkpoint inhibitor-based therapy as the first-line treatment for advanced non-small cell lung cancer: Efficacy, challenges, and future perspectives. Thorac Cancer. (2025) 16:e70113. doi: 10.1111/1759-7714.70113 40551477 PMC12185780

[10] Guo H Zhang J Qin C Yan H Luo X Zhou H . Advances and challenges of first-line immunotherapy for non-small cell lung cancer: A review. Medicine. (2024) 103:e36861. doi: 10.1097/md.0000000000036861 38241591 PMC10798763

[11] Mariniello A Borgeaud M Weiner M Frisone D Kim F Addeo A . Primary and acquired resistance to immunotherapy with checkpoint inhibitors in Nsclc: From bedside to bench and back. BioDrugs: Clin Immunother Biopharm Gene Ther. (2025) 39:215–35. doi: 10.1007/s40259-024-00700-2 39954220 PMC11906525

[12] Wang H Niu X Jin Z Zhang S Fan R Xiao H . Immunotherapy resistance in non-small cell lung cancer: From mechanisms to therapeutic opportunities. J Exp Clin Cancer Res: CR. (2025) 44:250. doi: 10.1186/s13046-025-03519-z 40849659 PMC12374485

[13] Hu X Rodday AM Gurinovich A Pan S Salei YV Lin JH . Real-world data of immune-related adverse events in lung cancer patients receiving immune-checkpoint inhibitors. Immunotherapy. (2025) 17:321–9. doi: 10.1080/1750743x.2025.2488728 40183219 PMC12045565

[14] Naidoo J Johnson DB Doran C Wang Y Zhang Y Le TK . Management of severe immune-related adverse events and outcomes in patients with advanced non-small cell lung cancer receiving immune checkpoint inhibitors. Oncologist. (2025) 30:1-10. doi: 10.1093/oncolo/oyae318 39566081 PMC12395253

[15] Gill GS Kharb S Goyal G Das P Kurdia KC Dhar R . Immune checkpoint inhibitors and immunosuppressive tumor microenvironment: Current challenges and strategies to overcome resistance. Immunopharmacol Immunotoxicol. (2025) 47:485–507. doi: 10.1080/08923973.2025.2504906 40376861

[16] Cabezón-Gutiérrez L Palka-Kotlowska M Custodio-Cabello S Chacón-Ovejero B Pacheco-Barcia V . Metabolic mechanisms of immunotherapy resistance. Explor Target Anti Tumor Ther. (2025) 6:1002297. doi: 10.37349/etat.2025.1002297 40092297 PMC11907103

[17] Ghani Khan K Kaur P Bhagat M Klair HK Bakhtawar M Aldea Saldaña JM . Effect of statin therapy on clinical outcomes in patients with cardiovascular risks: A systematic review and meta-analysis. Cureus. (2025) 17:e88238. doi: 10.7759/cureus.88238 40831859 PMC12359277

[18] Roberts CG Guallar E Rodriguez A . Efficacy and safety of statin monotherapy in older adults: A meta-analysis. J Gerontol Ser A Biol Sci Med Sci. (2007) 62:879–87. doi: 10.1093/gerona/62.8.879 17702880

[19] Khan S Huda B Bhurka F Patnaik R Banerjee Y . Molecular and immunomodulatory mechanisms of statins in inflammation and cancer therapeutics with emphasis on the Nf-Kb, Nlrp3 inflammasome, and cytokine regulatory axes. Int J Mol Sci. (2025) 26:8429. doi: 10.3390/ijms26178429 40943351 PMC12428962

[20] Zarei F Salimi Z Zeinivand M Zamani N Bahrani R . An overview of the antioxidant, anti-inflammatory, and anticancer effects of statins. Anti Cancer Agents Med Chem. (2026) 26(7):644–58. doi: 10.2174/0118715206377993251115050803 41588902

[21] Yang J Lin J Guo H Wu W Wang J Mao J . Administration of statins is correlated with favourable prognosis in lung cancer patients receiving immune checkpoint inhibitors. Front Immunol. (2025) 16:1638677. doi: 10.3389/fimmu.2025.1638677 41122174 PMC12535986

[22] Mao W Cai Y Chen D Jiang G Xu Y Chen R . Statin shapes inflamed tumor microenvironment and enhances immune checkpoint blockade in non-small cell lung cancer. JCI Insight. (2022) 7:e161940. doi: 10.1172/jci.insight.161940 35943796 PMC9675559

[23] Tilkin-Mariamé AF Cormary C Ferro N Sarrabayrouse G Lajoie-Mazenc I Faye JC . Geranylgeranyl transferase inhibition stimulates anti-melanoma immune response through Mhc class I and costimulatory molecule expression. FASEB J: Off Publ Fed Am Societies For Exp Biol. (2005) 19:1513–5. doi: 10.1096/fj.04-3482fje 15990392

[24] Ageta H Shimada Y Nagaoka T Takenaka K Yoshioka Y Konno K . Statins attenuate Pd-L1 sorting to small extracellular vesicles dependent on ubiquitin-like 3 modification. Sci Rep. (2025) 15:43802. doi: 10.1038/s41598-025-27789-x 41398009 PMC12706078

[25] Rossi A Filetti M Taurelli Salimbeni B Piras M Rizzo F Giusti R . Statins and immunotherapy: Togetherness makes strength the potential effect of statins on immunotherapy for Nsclc. Cancer Rep (Hoboken NJ). (2021) 4:e1368. doi: 10.1002/cnr2.1368 33788420 PMC8388159

[26] Svaton M Zemanova M Zemanova P Kultan J Fischer O Skrickova J . Impact of concomitant medication administered at the time of initiation of nivolumab therapy on outcome in non-small cell lung cancer. Anticancer Res. (2020) 40:2209–17. doi: 10.21873/anticanres.14182 32234916

[27] Takada K Shimokawa M Takamori S Shimamatsu S Hirai F Tagawa T . A propensity score-matched analysis of the impact of statin therapy on the outcomes of patients with non-small-cell lung cancer receiving anti-Pd-1 monotherapy: A multicenter retrospective study. BMC Cancer. (2022) 22:503. doi: 10.1186/s12885-022-09385-8 35524214 PMC9074359

[28] Cantini L Pecci F Hurkmans DP Belderbos RA Lanese A Copparoni C . High-intensity statins are associated with improved clinical activity of Pd-1 inhibitors in Malignant pleural mesothelioma and advanced non-small cell lung cancer patients. Eur J Cancer (Oxford Engl 1990). (2021) 144:41–8. doi: 10.1016/j.ejca.2020.10.031 33326868

[29] Liao Y Lin Y Ye X Shen J . Concomitant statin use and survival in patients with cancer on immune checkpoint inhibitors: A meta-analysis. JCO Oncol Pract. (2025) 21:989–1000. doi: 10.1200/op-24-00583 39772879

[30] Zhang L Wang H Tian J Sui L Chen X . Concomitant statins and the survival of patients with non-small-cell lung cancer treated with immune checkpoint inhibitors: A meta-analysis. Int J Clin Pract. (2022) 2022:3429462. doi: 10.1155/2022/3429462 35855055 PMC9276478

[31] Zhu Z Yuan X Zheng Y Dou B Liu L Loh PY . Effectiveness of acupuncture in managing aromatase inhibitor-related arthralgia in breast cancer: A systematic review and meta-analysis. Acupuncture Herbal Med. (2025) 5:352–65. doi: 10.1097/HM9.0000000000000172 42585464

[32] Zhang A Li J He T Xie H Mou X Yeung TC . Efficacy and safety of acupuncture in treating low back and pelvic girdle pain during pregnancy: A systematic review and meta-analysis of randomized controlled trials. Acupuncture Herbal Med. (2024) 4:346–57. doi: 10.1097/hm9.0000000000000093 42585464

[33] Callejo A Frigola J Iranzo P Carbonell C Diaz N Marmolejo D . Interrelations between patients' clinicopathological characteristics and their association with response to immunotherapy in a real-world cohort of Nsclc patients. Cancers (Basel). (2021) 13:3249. doi: 10.3390/cancers13133249 34209601 PMC8268100

[34] Cortellini A Di Maio M Nigro O Leonetti A Cortinovis DL Aerts JG . Differential influence of antibiotic therapy and other medications on oncological outcomes of patients with non-small cell lung cancer treated with first-line pembrolizumab versus cytotoxic chemotherapy. J Immunother Cancer. (2021) 9:e002421. doi: 10.1136/jitc-2021-002421 33827906 PMC8031700

[35] Kostine M Mauric E Tison A Barnetche T Barre A Nikolski M . Baseline co-medications may alter the anti-tumoural effect of checkpoint inhibitors as well as the risk of immune-related adverse events. Eur J Cancer (Oxford Engl 1990). (2021) 157:474–84. doi: 10.1016/j.ejca.2021.08.036 34649118

[36] Marrone MT Reuss JE Crawford A Neelon B Liu JO Brahmer JR . Statin use with immune checkpoint inhibitors and survival in nonsmall cell lung cancer. Clin Lung Cancer. (2025) 26:201–9. doi: 10.1016/j.cllc.2024.12.008 39818516 PMC12037305

[37] Miura K Sano Y Niho S Kawasumi K Mochizuki N Yoh K . Impact of concomitant medication on clinical outcomes in patients with advanced non-small cell lung cancer treated with immune checkpoint inhibitors: A retrospective study. Thorac Cancer. (2021) 12:1983–94. doi: 10.1111/1759-7714.14001 33990133 PMC8258365

[38] Omori M Okuma Y Hakozaki T Hosomi Y . Statins improve survival in patients previously treated with nivolumab for advanced non-small cell lung cancer: An observational study. Mol Clin Oncol. (2019) 10:137–43. doi: 10.3892/mco.2018.1765 30655989 PMC6313973

[39] Yakobson A Agbarya A Dudnik Y Gothelf I Miari A Brenner R . Impact of statin use on immunotherapy outcomes and efficacy in non-small cell lung cancer patients. Int J Mol Sci. (2026) 27:1541. doi: 10.3390/ijms27031541 41683960 PMC12898650

[40] Song J Chi KY Jeon H Chang YC Xanthavanij N Tang Z . Statin and immune-related cardiovascular events in lung cancer patients receiving immune checkpoint inhibitors. Oncology. (2026) 104:319–26. doi: 10.1159/000546204 40398400

[41] Abdelhamid A Nasrallah D Ahmed E Sahebkar A Eid AH . Beyond cardiovascular health: The pharmacotherapeutic potential of statins in oncology. Semin Cancer Biol. (2025) 117:1–22. doi: 10.1016/j.semcancer.2025.10.003 41614590

[42] Su J Ji C Niu X Wu Z Shi L Kang L . Atorvastatin as a pleiotropic anticancer agent: Mechanisms, evidence, and therapeutic repurposing potential. Front Immunol. (2026) 17:1808729. doi: 10.3389/fimmu.2026.1808729 42112329 PMC13153102

[43] Talty R Brooks VT McGeary MK Milette S Zheng S Flem-Karlsen K . Lipophilic statins deplete Gpx4 to promote ferroptosis and sensitize cancer cells to immune checkpoint blockade. Mol Cancer Ther. (2026) 25:759–70. doi: 10.1158/1535-7163.mct-25-0667 41423587 PMC12752129

[44] Liu W Wang Z Li Z Li S Shi X Xu Y . Lipid metabolic reprogramming in the tumor microenvironment and its mechanistic role in immunosuppressive cells. Front Immunol. (2025) 16:1728354. doi: 10.3389/fimmu.2025.1728354 41312365 PMC12647040

[45] Lagunas-Rangel FA . Cholesterol effects on the tumor immune microenvironment: From fundamental concepts to mechanisms and implications. Front Oncol. (2025) 15:1579054. doi: 10.3389/fonc.2025.1579054 40270603 PMC12014580

[46] Sun D Cui X Yang W Wei M Yan Z Zhang M . Simvastatin inhibits Pd-L1 via Ilf3 to induce ferroptosis in gastric cancer cells. Cell Death Dis. (2025) 16:208. doi: 10.1038/s41419-025-07562-8 40140647 PMC11947124

[47] Sabeel S Motaung B Nguyen KA Ozturk M Mukasa SL Wolmarans K . Impact of statins as immune-modulatory agents on inflammatory markers in adults with chronic diseases: A systematic review and meta-analysis. PloS One. (2025) 20:e0323749. doi: 10.1371/journal.pone.0323749 40440323 PMC12121830

