# Supplementary material for: Statins use and prognosis in lung cancer patients treated with immune checkpoint inhibitors: evidence from a meta-analysis
Source: Front Immunol. 2026 Sep 1;17:1910678. doi: 10.3389/fimmu.2026.1910678 (PMC13575920; doi:10.3389/fimmu.2026.1910678)
Supplement: Supplementary file 1 [file Table1.docx]

| **Supplementary Materials Table S1. Search strategy** | |
| --- | --- |
| Pubmed | ("Hydroxymethylglutaryl-CoA Reductase Inhibitors"[MeSH] OR "Statin"[Title/Abstract] OR "Statins"[Title/Abstract] OR "Inhibitors, HMG-CoA Reductase"[Title/Abstract] OR "Inhibitors, HMG CoA Reductase"[Title/Abstract] OR "Reductase Inhibitors, HMG-CoA"[Title/Abstract] OR "HMG-CoA Reductase Inhibitors"[Title/Abstract] OR "HMG CoA Reductase Inhibitors"[Title/Abstract] OR "Inhibitors, Hydroxymethylglutaryl-Coenzyme A"[Title/Abstract] OR "Hydroxymethylglutaryl-Coenzyme A Inhibitors"[Title/Abstract] OR "Inhibitors, Hydroxymethylglutaryl Coenzyme A"[Title/Abstract] OR "Inhibitors, Hydroxymethylglutaryl-CoA"[Title/Abstract] OR "Hydroxymethylglutaryl-CoA Inhibitors"[Title/Abstract] OR "Inhibitors, Hydroxymethylglutaryl CoA"[Title/Abstract] OR "Hydroxymethylglutaryl-CoA Reductase Inhibitor"[Title/Abstract] OR "Hydroxymethylglutaryl CoA Reductase Inhibitor"[Title/Abstract] OR "Reductase Inhibitor,Hydroxymethylglutaryl-CoA"[Title/Abstract] OR "Statins, HMG-CoA"[Title/Abstract] OR "HMG-CoA Statins"[Title/Abstract] OR "Statins, HMG CoA"[Title/Abstract] OR "Atorvastatin"[Title/Abstract] OR "Simvastatin"[Title/Abstract] OR "Rosuvastatin"[Title/Abstract] OR "Pravastatin"[Title/Abstract] OR "Pivalatedstatin"[Title/Abstract] OR "Fluvastatin"[Title/Abstract] OR "Lovastatin"[Title/Abstract]) AND ("Immune Checkpoint Inhibitors"[MeSH] OR "Checkpoint Inhibitors, Immune"[Title/Abstract] OR "Immune Checkpoint Blockers"[Title/Abstract] OR "Checkpoint Blockers, Immune"[Title/Abstract] OR "Immune Checkpoint Inhibitor"[Title/Abstract] OR "Checkpoint Inhibitor, Immune"[Title/Abstract] OR "CTLA-4 Inhibitors"[Title/Abstract] OR "CTLA 4 Inhibitors"[Title/Abstract] OR "Cytotoxic T-Lymphocyte-Associated Protein 4 Inhibitors"[Title/Abstract] OR "Cytotoxic T Lymphocyte Associated Protein 4 Inhibitors"[Title/Abstract] OR "Cytotoxic T-Lymphocyte-Associated Protein 4 Inhibitor"[Title/Abstract] OR "Cytotoxic T Lymphocyte Associated Protein 4 Inhibitor"[Title/Abstract] OR "CTLA-4 Inhibitor"[Title/Abstract] OR "CTLA 4 Inhibitor"[Title/Abstract] OR "PD-1 Inhibitors"[Title/Abstract] OR "PD 1 Inhibitors"[Title/Abstract] OR "Programmed Cell Death Protein 1 Inhibitor"[Title/Abstract] OR "Programmed Cell Death Protein 1 Inhibitors"[Title/Abstract] OR "PD-1 Inhibitor"[Title/Abstract] OR "Inhibitor, PD-1"[Title/Abstract] OR "PD 1 Inhibitor"[Title/Abstract] OR "Immune Checkpoint Blockade"[Title/Abstract] OR "Checkpoint Blockade, Immune"[Title/Abstract] OR "Immune Checkpoint Inhibition"[Title/Abstract] OR "Checkpoint Inhibition, Immune"[Title/Abstract] OR "PD-L1 Inhibitors"[Title/Abstract] OR "PD L1 Inhibitors"[Title/Abstract] OR "Programmed Death-Ligand 1 Inhibitors"[Title/Abstract] OR "Programmed Death Ligand 1 Inhibitors"[Title/Abstract] OR "PD-L1 Inhibitor"[Title/Abstract] OR "PD L1 Inhibitor"[Title/Abstract] OR "PD-1-PD-L1 Blockade"[Title/Abstract] OR "Blockade, PD-1-PD-L1"[Title/Abstract] OR "PD 1 PD L1 Blockade"[Title/Abstract] OR "Pembrolizumab"[Title/Abstract] OR "Nivolumab"[Title/Abstract] OR "Atezolizumab"[Title/Abstract] OR "Ipilimumab"[Title/Abstract] OR "Avelumab"[Title/Abstract] OR "Tremelimumab"[Title/Abstract] OR "Durvalumab"[Title/Abstract] OR "Cemiplimab"[Title/Abstract] OR "Sintilimab"[Title/Abstract] OR "Camrelizumab"[Title/Abstract] OR "Dostarlimab"[Title/Abstract] OR "Tislelizumab"[Title/Abstract] OR "Penpulimab"[Title/Abstract] OR "Toripalimab"[Title/Abstract] OR "Zimberelimab"[Title/Abstract] OR "Serplulimab"[Title/Abstract] OR "Pucotenlimab"[Title/Abstract] OR "Envafolimab"[Title/Abstract] OR "Sugemalimab"[Title/Abstract]) AND("Lung Neoplasms"[MeSH] OR "Carcinoma, Non-Small-Cell Lung"[MeSH] OR "Carcinoma, Small Cell"[MeSH]) OR ("lung cancer"[Title/Abstract] OR "lung cancers"[Title/Abstract] OR "lung carcinoma"[Title/Abstract] OR "lung carcinomas"[Title/Abstract] OR "lung neoplasm"[Title/Abstract] OR "lung neoplasms"[Title/Abstract] OR "pulmonary cancer"[Title/Abstract] OR "pulmonary carcinoma"[Title/Abstract] OR "cancer of the lung"[Title/Abstract] OR "cancer of lung"[Title/Abstract] OR "non-small cell lung cancer"[Title/Abstract] OR "non-small cell lung carcinoma"[Title/Abstract] OR "NSCLC"[Title/Abstract] OR "non small cell lung cancer"[Title/Abstract] OR "non small cell lung carcinoma"[Title/Abstract] OR "non-small-cell lung carcinoma"[Title/Abstract] OR "small cell lung cancer"[Title/Abstract] OR "small cell lung carcinoma"[Title/Abstract] OR "SCLC"[Title/Abstract] OR "small cell cancer of the lung"[Title/Abstract] OR "oat cell lung cancer"[Title/Abstract] OR "oat cell carcinoma"[Title/Abstract]) AND ("survival"[Title/Abstract] OR "overall survival"[Title/Abstract] OR "progression-free survival"[Title/Abstract] OR "mortality"[Title/Abstract] OR "survival rate"[Title/Abstract] OR "survival analysis"[Title/Abstract] OR "prognosis"[Title/Abstract] OR "death"[Title/Abstract]) |
| The Cochrane Library | #1 MeSH descriptor: [Hydroxymethylglutaryl-CoA Reductase Inhibitors] explode all trees #2 (statin* or atorvastatin or simvastatin or rosuvastatin or pravastatin or pivalatedstatin or fluvastatin or lovastatin):ti,ab,kw OR ("HMG-CoA reductase inhibitor*" or "HMG CoA reductase inhibitor*" or "hydroxymethylglutaryl-CoA reductase inhibitor*"):ti,ab,kw #3 #1 or #2 #4 MeSH descriptor: [Immune Checkpoint Inhibitors] explode all trees #5 ("immune checkpoint inhibitor*" or "CTLA-4 inhibitor*" or "CTLA4 inhibitor*" or "PD-1 inhibitor*" or "PD1 inhibitor*" or "PD-L1 inhibitor*" or "PDL1 inhibitor*" or "immune checkpoint blockade" or "immune checkpoint inhibition"):ti,ab,kw #6 (pembrolizumab or nivolumab or atezolizumab or ipilimumab or avelumab or tremelimumab or durvalumab or cemiplimab or sintilimab or camrelizumab or dostarlimab or tislelizumab or penpulimab or toripalimab or zimberelimab or serplulimab or pucotenlimab or envafolimab or sugemalimab):ti,ab,kw #7 #4 or #5 or #6 #8 MeSH descriptor: ["Carcinoma, Non-Small-Cell Lung" or "Lung Neoplasms"  or "Carcinoma, Small Cell"] explode all trees #9 ("lung cancer" or "lung cancers" or "lung carcinoma" or "lung carcinomas" or "lung neoplasm" or "lung neoplasms" or "pulmonary cancer" or "pulmonary carcinoma" or  "cancer of the lung" or "cancer of lung" or "non-small cell lung cancer" or "non-small cell lung carcinoma" or "NSCLC" or "non small cell lung cancer" or "non small cell lung carcinoma" or "non-small-cell lung carcinoma" or "small cell lung cancer" or "small cell lung carcinoma" or "SCLC" or "small cell cancer of the lung" or "oat cell lung cancer" or "oat cell carcinoma"):ti,ab,kw #10 #8 or #9 #11 (survival or "overall survival" or "progression-free survival" or mortality or "survival rate" or "survival analysis" or prognosis or death):ti,ab,kw #12 #3 and #7 and #10 and #11 |
| Embase | #1 'hydroxymethylglutaryl coenzyme A reductase inhibitor'/exp #2 'statin'/exp #3 statin*:ti,ab,kw OR 'hydroxymethylglutaryl coenzyme A reductase inhibitor*':ti,ab,kw OR 'hmg coa reductase inhibitor*':ti,ab,kw OR 'hmg-coa reductase inhibitor*':ti,ab,kw #4 atorvastatin:ti,ab,kw OR simvastatin:ti,ab,kw OR rosuvastatin:ti,ab,kw OR pravastatin:ti,ab,kw OR pivalatedstatin:ti,ab,kw OR fluvastatin:ti,ab,kw OR lovastatin:ti,ab,kw #5 #1 OR #2 OR #3 OR #4 #6 'immune checkpoint inhibitor'/exp #7 'pd-1 inhibitor'/exp OR 'pd-l1 inhibitor'/exp OR 'ctla-4 inhibitor'/exp #8 'immune checkpoint inhibitor*':ti,ab,kw OR 'checkpoint inhibitor*':ti,ab,kw OR 'ctla-4 inhibitor*':ti,ab,kw OR 'pd-1 inhibitor*':ti,ab,kw OR 'pd-l1 inhibitor*':ti,ab,kw OR 'immune checkpoint blockade':ti,ab,kw OR 'immune checkpoint inhibition':ti,ab,kw #9 pembrolizumab:ti,ab,kw OR nivolumab:ti,ab,kw OR atezolizumab:ti,ab,kw OR ipilimumab:ti,ab,kw OR avelumab:ti,ab,kw OR tremelimumab:ti,ab,kw OR durvalumab:ti,ab,kw OR cemiplimab:ti,ab,kw OR sintilimab:ti,ab,kw OR camrelizumab:ti,ab,kw OR dostarlimab:ti,ab,kw OR tislelizumab:ti,ab,kw OR penpulimab:ti,ab,kw OR toripalimab:ti,ab,kw OR zimberelimab:ti,ab,kw OR serplulimab:ti,ab,kw OR pucotenlimab:ti,ab,kw OR envafolimab:ti,ab,kw OR sugemalimab:ti,ab,kw #10 #6 OR #7 OR #8 OR #9 #11 'lung cancer'/exp OR 'non small cell lung cancer'/exp OR 'small cell lung cancer'/exp OR 'lung tumor'/exp OR 'lung carcinoma'/exp OR 'small cell carcinoma'/exp #12 'lung cancer':ab,ti,kw OR 'lung cancers':ab,ti,kw OR 'lung carcinoma':ab,ti,kw OR 'lung carcinomas':ab,ti,kw OR 'lung neoplasm':ab,ti,kw OR 'lung neoplasms':ab,ti,kw OR 'pulmonary cancer':ab,ti,kw OR 'pulmonary carcinoma':ab,ti,kw OR 'cancer of the lung':ab,ti,kw OR 'cancer of lung':ab,ti,kw OR 'non-small cell lung cancer':ab,ti,kw OR 'non-small cell lung carcinoma':ab,ti,kw OR 'NSCLC':ab,ti,kw OR 'non small cell lung cancer':ab,ti,kw OR 'non small cell lung carcinoma':ab,ti,kw OR 'non-small-cell lung carcinoma':ab,ti,kw OR 'small cell lung cancer':ab,ti,kw OR 'small cell lung carcinoma':ab,ti,kw OR 'SCLC':ab,ti,kw OR 'small cell cancer of the lung':ab,ti,kw OR 'oat cell lung cancer':ab,ti,kw OR 'oat cell carcinoma':ab,ti,kw #13 #11 OR #12 #14 'survival'/exp OR 'overall survival'/exp OR 'progression free survival'/exp OR 'survival rate'/exp #15 survival:ti,ab,kw OR 'overall survival':ti,ab,kw OR 'progression-free survival':ti,ab,kw OR mortality:ti,ab,kw OR 'survival rate':ti,ab,kw OR 'survival analysis':ti,ab,kw OR prognosis:ti,ab,kw OR death:ti,ab,kw #16 #14 OR #15 #17 #5 AND #10 AND #13 AND #16 #18 #17 AND [humans]/lim |
| Web of Science | TS=((statin* OR "hydroxymethylglutaryl-CoA reductase inhibitor*" OR "HMG-CoA reductase inhibitor*" OR "HMG CoA reductase inhibitor*" OR atorvastatin OR simvastatin OR rosuvastatin OR pravastatin OR pivalatedstatin OR fluvastatin OR lovastatin) AND ("immune checkpoint inhibitor*" OR "CTLA-4 inhibitor*" OR "PD-1 inhibitor*" OR "PD-L1 inhibitor*" OR "immune checkpoint blockade" OR "immune checkpoint inhibition" OR pembrolizumab OR nivolumab OR atezolizumab OR ipilimumab OR avelumab OR tremelimumab OR durvalumab OR cemiplimab OR sintilimab OR camrelizumab OR dostarlimab OR tislelizumab OR penpulimab OR toripalimab OR zimberelimab OR serplulimab OR pucotenlimab OR envafolimab OR sugemalimab) AND ("lung cancer" OR "lung cancers" OR "lung carcinoma" OR "lung carcinomas" OR "lung neoplasm" OR "lung neoplasms" OR "pulmonary cancer" OR "pulmonary carcinoma" OR "cancer of the lung" OR "cancer of lung" OR "non-small cell lung cancer" OR "non-small cell lung carcinoma" OR "non small cell lung cancer" OR "non small cell lung carcinoma" OR "non-small-cell lung carcinoma" OR "NSCLC" OR "small cell lung cancer" OR "small cell lung carcinoma" OR "SCLC" OR "small cell cancer of the lung" OR "oat cell lung cancer" OR "oat cell carcinoma"  ) AND (survival OR "overall survival" OR "progression-free survival" OR mortality OR "survival rate" OR "survival analysis" OR prognosis OR death)) |
